# Recommended nutrition-related practices for online food delivery companies

**DOI:** 10.1017/S1368980023002495

**Published:** 2023-12

**Authors:** Lana Vanderlee, Gary Sacks

**Affiliations:** 1 École de Nutrition, Centre NUTRISS (Nutrition, Santé et Société), Institute of Nutrition and Functional Foods (INAF), Université Laval, 2440 Boulevard Hochelaga, Quebec, QC G1V 0A6, Canada; 2 Deakin University, Institute for Health Transformation, Global Centre for Preventive Health and Nutrition, Geelong, VIC, Australia

**Keywords:** Online food delivery, Public policy, Digital food environment, Nutrition policy, Food away from home

The online food delivery (OFD) industry has realised considerable growth over the last decade. In 2023, overall revenue in the global OFD market is forecasted to be over USD1 trillion, with future growth rates of over 10 % per year expected^(1)^. While few empirical studies have examined patterns of use, a multi-country study from 2018 estimated that 15 % of consumers had used OFD in the past 7 d, with higher use in particular population groups, such as younger adults and those living with children^(2)^.

From a public health perspective, OFD has been identified as a potential contributor to unhealthy diets and a threat to the achievement of the UN Sustainable Development Goals^(3,4)^. Firstly, the rise in OFD has increased the availability and ease of access to food prepared away from home (FAFH)^(5,6)^, which is known to be associated with poorer quality diets and higher BMI^(7,8)^. Secondly, concerns have been raised about the role of OFD companies in consolidating power in the restaurant industry, including amongst the most dominant fast-food chains^(9,10)^, which may contribute to diet-related disease and health inequity more generally^(11)^.

At the same time, the increased prominence of OFD presents an opportunity for public health. OFD companies could, theoretically, promote consumption of healthier FAFH, and incentivise restaurant companies to adopt practices that are health-promoting^(12–14)^. At present, there is limited regulation of OFD practices related to nutrition and public health and limited monitoring of OFD practices from a nutrition perspective^(4,12,15)^.

In this commentary, we propose recommended nutrition-related practices for OFD companies, based on a previously applied framework, the BIA-Obesity (Business Impact Assessment – Obesity and population nutrition) tool, for assessing the nutrition-related commitments and actions of food companies in various sectors (including manufacturers, retailers and restaurants)^(16)^. We contextualised the indicators in the BIA-Obesity tool to the OFD setting, taking into account extant peer-reviewed and grey literature regarding best practice and recommended industry actions. We categorised recommended policies and actions across five domains: *corporate strategy; nutrition information; promotions and pricing; product and outlet availability;* and *product formulation.* A summary of the recommendations can be found in Table 1.

**Table 1:** Summary of recommended nutrition-related policies and actions for online food delivery companies

Domain	Global recommendation	Specific actions
*Corporate strategy*	*Develop an overall strategy related to nutrition and health*	• As part of the overall corporate strategy and mission statement, recognise the role the company plays in efforts to improve population diets and achieve the Sustainable Development Goals • Establish targets for the proportion of revenue generated from healthier restaurants and the proportion of healthy food sold on platforms • Implement routine reporting on a range of nutrition-related metrics (e.g. overall nutrient profile of products available for sale, provision of nutrition information), including progress against commitments and targets • Provide transparency related to nutrition-related algorithms and practices • Transparently report relationships with external groups (including governments, industry groups, universities and community organisations) and policy positions (e.g. related to major policy issues, such as nutrition labelling and food-related taxes) • Limit political lobbying and donations
*Nutrition information*	*Provide nutrition information in a consistent manner that is easily and predictably available to consumers*	• Ensure that vendors selling products on their platforms are meeting existing government nutrition labelling regulations (e.g. related to provision of information about energy content and associated contextual statements) • For all items available for sale, provide both detailed (e.g. ingredients lists, nutrition information panels) and summary nutrition information in a consistent manner that is easily and predictably available to consumers in prominent ways (e.g. summary information next to the item name or price, in equivalent size) • Publish clear criteria for the use of symbols, descriptors and logos that have health connotations (such as logos depicting ‘healthy’ items or ‘healthy’ restaurants) that are in line with locally relevant government-led programmes or policies, when available • Provide tools, such as automated nutrition calculators, that help consumers track and understand the nutritional composition of orders placed, and routinely evaluate their effectiveness from a nutritional perspective.
*Promotions and pricing*	*Restrict the exposure of children (under 18 years of age) to the marketing of unhealthy foods and related brands*	• Restrict promotions that feature unhealthy products and related brands, and only enter into joint marketing campaigns with restaurants that have healthy product profiles overall • Avoid promotion techniques, such as partnerships with celebrities, characters or influencers, that appeal to young people • Avoid sponsorships or branding related to settings and events popular with children and families, including sports events • Limit premium offers and price discounts (including promotion codes, loyalty discounts, and free delivery offers) for unhealthy products and brands, and use premium offers exclusively to incentivise healthier selections • Adopt transparency regarding methods (and related algorithms) used for target marketing (e.g. to particular population groups and individual characteristics) • Responsibly use consumer data so as not to inequitably target vulnerable groups
*Product and outlet availability*	*Increase the visibility and accessibility of healthier products on OFD platforms*	• Prioritise the position and presentation of healthier options on landing pages and within search results, actively promoting healthier options • Ensure vendors make healthier options, such as low or no-calorie beverages and salad or fresh vegetables, the default in meal combinations (e.g. children’s meals) • Limit the selection of ‘add-on’ options to healthier products • Reduce barriers to entry into the OFD market, and support increased participation from smaller restaurant companies
*Product formulation*	*Incentivise development of healthier foods/menu offerings from vendors using OFD platforms*	• Develop clear, consistent and transparent criteria for determining and communicating the healthfulness of individual foods, brands and restaurants, based on government-endorsed guidelines and/or nutrient profiling schemes • Set goals for the proportion of healthier restaurants on each platform • Prioritise the presentation of healthier restaurants within existing structures • Offer incentives to suppliers for making product improvements (e.g. use of healthier frying oils)

OFD, online food delivery.

## Overview of how the online food delivery industry works

The OFD industry is dominated by a small group of OFD aggregators (e.g. Uber Eats, DoorDash) that provide online platforms that link consumers to food available for delivery or pickup within their geographic area^(4)^. OFD companies act as third-party intermediaries, with restaurant companies able to elect to make their products available through particular platforms for a fee, often a percentage of each order, with additional fees to improve marketing opportunities (e.g., prioritised placement, improved appearance in search results) within the platform. Restaurants are responsible for entering information into the platform’s ‘back end’ related to the products on offer, prices and the use of some symbols or logos for a product (such as ‘vegetarian’ and/or ‘healthy’). In addition, a restaurant chooses descriptors (i.e. restaurant types) under which the restaurant will be categorised (e.g. ‘sandwich’, ‘healthy’, ‘Asian’). At present, it seems as though the provision of nutrition information on OFD platforms is provided at the discretion of the restaurateur. The selection of restaurants available to a consumer on an OFD platform is based on a range of factors, including their proximity to restaurant outlets, outlet operating hours and availability of delivery drivers. OFD platforms use proprietary algorithms to tailor the list of available restaurants to individual consumers based on marketing arrangements with restaurants, and using machine learning techniques that draw on customer characteristics and previous purchase behaviour^(17)^.

## Corporate strategy

In line with nutrition-related recommendations for all major food companies, we recommend that major OFD companies include public commitments to nutrition and health as part of their corporate strategies, with a focus on targets related to the proportion of revenue generated from healthier restaurants and/or healthier food items. Such commitments can provide an overarching framework to guide company policies and actions. We also recommend that OFD companies routinely report on a range of nutrition-related metrics, including actions taken and progress against commitments and targets. Furthermore, we recommend that OFD companies are transparent about their external relationships (e.g. with governments and community groups) and policy positions (e.g., related to major policy issues, such as nutrition labelling and food-related taxes). Best practice in this area includes policies that limit corporate lobbying and political donations.

## Nutrition information

In the area of nutrition information, OFD companies have an obligation to ensure that vendors selling products on their platforms are meeting existing government regulations (e.g. related to the provision of information about energy content). Empirical research suggests that, in some contexts, obligations are not being met^(18,19)^. We recommend that OFD companies ensure that their platforms provide both detailed and summary nutrition information for all items available for sale and facilitate restaurants to provide this information in a consistent manner that is easily and predictably available to consumers in prominent ways.

If OFD companies are using symbols, descriptors or logos that have health connotations, clear and transparent criteria are required and should align with and support government-led programmes or policies when possible. For example, OFD companies could help promote the provision of country-specific interpretative nutrition indicators, such as traffic lights, health star ratings or ‘high in’ symbols, that are based on government-endorsed criteria. We recommend that OFD companies also provide tools, such as automated nutrition calculators, that help consumers track and understand the nutritional composition of orders placed. Use of such tools and their effectiveness from a nutrition perspective should be routinely evaluated^(20)^.

## Promotions and pricing

In line with WHO recommendations^(21,22)^, we recommend that OFD companies restrict the exposure of children (under 18 years of age) to the marketing of unhealthy foods and related brands. Given the strong association of FAFH consumption and unhealthy diets^(8)^, and the predominance of unhealthy fast-food chains on most major OFD platforms^(9,23)^, restrictions likely need to apply comprehensively across a range of promotion strategies used by OFD companies. In particular, we recommend that OFD companies avoid promotion techniques, such as partnerships with celebrities, that appeal to young people, as well as sponsorships and branding related to settings and events popular with children and families. In addition, we recommend OFD companies restrict promotions that feature unhealthy products and related brands and only enter into joint marketing campaigns with restaurants that have healthy product profiles overall.

Comprehensive actions from OFD companies to restrict unhealthy food marketing would extend to a commitment to avoid the use of premium offers or price discounts for unhealthy products and brands. Instead, we recommend that OFD companies use price-related promotions exclusively to incentivise healthier selections.

More broadly in the area of marketing, and in recognition of emerging issues related to data privacy and digital marketing^(21)^ and concerns regarding the way in which particular population groups are targeted by the marketing strategies of food companies^(24)^, we recommend that OFD companies commit to transparency in the methods used for target marketing, and responsible use of consumer data, in line with relevant regulations.

## Product and outlet availability

We recommend that OFD companies increase the visibility and accessibility of healthier products on their platforms. Choice architecture in online food environments has been identified as an opportunity to nudge consumers in a healthier direction^(14,25)^. OFD companies can do this in a range of ways, such as prioritising the position and presentation of healthier options on landing pages and within search results. In addition, OFD companies can actively promote healthier product selection by ensuring vendors make healthier options, such as low or no-calorie beverages and salad or fresh vegetables, the default in meal combinations (e.g. children’s meals). OFD companies can also promote healthier purchases by limiting the selection of ‘add-on’ options (e.g. a prompt of ‘Would you like to add…’) to healthier products.

Lastly, OFD companies can take steps to encourage greater diversity of restaurant participation in their platforms, which may reduce the dominance of unhealthy fast-food chains and support the development of healthier food systems. In this area, we recommend that OFD companies adopt policies and practices that reduce barriers to entry into the OFD market and support increased participation from smaller restaurant companies.

## Product formulation

Many of the recommendations described in earlier sections are likely to encourage restaurants to increase the healthfulness of their offerings. OFD companies could also directly incentivise restaurants to offer healthier products, by requiring restaurants to use healthier frying oils or to meet category-specific nutrient targets (e.g. in relation to sodium and sugar), as a pre-condition to being listed on the platform. We recommend that all company actions in this area are underpinned by clear and transparent criteria for assessing the healthfulness of foods and restaurants, based on government-endorsed guidelines and/or nutrient profiling schemes. We recommend that OFD companies set goals for the proportion of healthier restaurants on their platform and prioritise the presentation of healthier restaurants within existing structures.

## Towards health-promoting online food delivery environments

In this commentary, we have identified a range of actions that OFD companies can take to contribute to efforts to improve population diets. While the recommendations are primarily directed towards OFD aggregators, we recognise that their ability to provide healthier foods is largely dependent on the practices and products offered by the restaurants selling products on their platforms. Accordingly, improvements to the healthfulness of FAFH will rely on restaurant companies improving the healthfulness of their food items and the way in which they are marketed. Critically, given the substantial market power that major OFD companies hold and their contractual agreements with restaurants, OFD companies have a substantial opportunity to influence the practices of restaurants related to nutrition.

Despite limited monitoring of the OFD sector related to nutrition, it is clear that very few of the recommended actions have been implemented in practice. While the field is growing rapidly and OFD company practices are likely to evolve quickly, experience from other areas of the food industry shows that voluntary actions in the area of nutrition often fall far short of recommendations^(26–28)^. As such, it is likely that government regulation will be needed to establish a ‘level playing field’ for all companies and ensure that online food environments are health-promoting. Apart from limited regulations related to menu labelling in some jurisdictions, there is currently a lack of nutrition-related regulations that apply in the FAFH space. The recommendations proposed in this commentary may help identify areas for regulation, including ways in which existing regulations (and approaches to compliance monitoring and enforcement) may need to be adapted for application to online food environments^(18,19)^.

Importantly, some of the recommended actions for OFD companies, such as prioritising healthier options within proprietary algorithms, may prove challenging to regulate. In these cases, OFD companies are only likely to be driven to change by consumer demand, actions by competitors and/or pressure from other stakeholders (such as public health groups, the media and investors)^(20,29)^. OFD companies can take steps towards the implementation of the recommendations by focusing on giving their customers more autonomy in shaping their online food environment. For example, OFD users could be given a choice to prioritise healthier restaurants or meals within their own algorithms, to apply filters to include only healthier restaurants and/or options or to limit the promotions related to less healthful foods to which they are exposed. Several of the recommendations we have made rely on a clear definition of healthy foods and brands. While multiple systems and approaches have been identified for classifying the healthfulness of FAFH and restaurants^(30–34)^, the strengths and weaknesses of applying existing nutrient profiling algorithms in this space, and the applicability of existing systems in diverse contexts are not well established and should be the focus of future research. Lastly, while this commentary has focused on nutrition, the rise of OFD has been noted as having potential negative impacts on other aspects of health and society, including environmental sustainability and workers’ rights^(3,4,15,35,36)^. Future research should consider integrating these considerations into a broader set of recommendations for the sector.
